# HDTD: analyzing multi-tissue gene expression data

**DOI:** 10.1093/bioinformatics/btw224

**Published:** 2016-06-07

**Authors:** Anestis Touloumis, John C. Marioni, Simon Tavaré

**Affiliations:** ^1^CRUK Cambridge Institute, University of Cambridge, Li Ka Shing Centre, Robinson Way, Cambridge CB2 0RE, UK; ^2^Computing, Engineering and Mathematics, University of Brighton, Brighton BN2 4GJ, UK; ^3^EMBL-European Bioinformatics Institute, Wellcome Genome Campus, Hinxton, Cambridge CB10 1SD, UK

## Abstract

**Motivation**: By collecting multiple samples per subject, researchers can characterize intra-subject variation using physiologically relevant measurements such as gene expression profiling. This can yield important insights into fundamental biological questions ranging from cell type identity to tumour development. For each subject, the data measurements can be written as a matrix with the different subsamples (e.g. multiple tissues) indexing the columns and the genes indexing the rows. In this context, neither the genes nor the tissues are expected to be independent and straightforward application of traditional statistical methods that ignore this two-way dependence might lead to erroneous conclusions. Herein, we present a suite of tools embedded within the R/Bioconductor package *HDTD* for robustly estimating and performing hypothesis tests about the mean relationship and the covariance structure within the rows and columns. We illustrate the utility of *HDTD* by applying it to analyze data generated by the Genotype-Tissue Expression consortium.

**Availability and Implementation**: The R package *HDTD* is part of Bioconductor. The source code and a comprehensive user’s guide are available at http://bioconductor.org/packages/release/bioc/html/HDTD.html.

**Contact**: A.Touloumis@brighton.ac.uk

**Supplementary information:** Supplementary data are available at *Bioinformatics* online.

## 1 Introduction

The term ‘transposable data’ refers to data that are naturally written in a matrix whose dimensions correspond to two distinct features of interest, while the term ‘high-dimensional’ reflects the fact that the dimension of the subject-specific data matrix is larger than the number of subjects. High-dimensional transposable data can be found in genetics, e.g. when, for each subject, gene expression levels are measured in multiple tissues (Piccirillo *et al.*, 2015), in different fragments of the same tumour (Sottoriva *et al.*, 2013) or in a well-defined spatial order (Petretto *et al.*, 2010), in yeast expression studies (Smith and Kruglyak, 2008), in protein-signaling networks (Sachs *et al.*, 2005), in eQTL analysis (Bhadra and Mallick, 2013) and in other studies with EEG, fMRI and time-series data (cf. Touloumis *et al.*, 2014). To analyze robustly such datasets, we developed the R package *HDTD* (High-Dimensional Transposable Data).

In multiple-tissue gene expression studies, the rows correspond to genes and the columns to tissues, and genes and tissues might to be correlated with each other. Ignoring a potential tissue-wise correlation could be misleading in determining the strength of the gene-wise correlation (Touloumis *et al.*, 2014) and it may hinder the discovery of differentially expressed genes, since traditional ANOVA-type tests suffer from extremely low power and/or false positive findings (Touloumis *et al.*, 2015). The unique feature of *HDTD* is the implementation of sound statistical methods that account for and estimate both the tissue- and gene-wise correlation, thus facilitating reliable inference about the form of the mean gene expression levels and the functional relationship among genes and/or tissues.

## 2 Statistical background

To introduce the notation, suppose that the gene expression levels for subject *i* are recorded in an *r *×* c* matrix $X_{i}$ with rows the same set of *r* genes and columns the same set of *c* tissues. We assume that $X_{1},\ldots ,X_{N}$ are independently and identically distributed. Inference about the mean relationship of the genes across the tissues and about the dependence structure relies on estimating and/or testing hypotheses about the mean matrix ***M***, the gene covariance matrix $\Sigma_{\text{R}}$ and the tissue covariance matrix $\Sigma_{\text{C}}$. In particular, the (*a*, *b*) element of ***M*** determines the mean expression level for gene *a* in tissue *b*, the (*c*, *d*) element of $\Sigma_{\text{R}}$ the covariance of genes *c* and *d*, and the (*e*, *f*) element of $\Sigma_{\text{C}}$ the covariance of tissues *e* and *f*. The covariance structure between two elements of a typical ***X*** has a Kronecker product form: $\text{Cov}(X_{ij},X_{lm})=\Sigma_{\text{R}il}\;\Sigma_{\text{C}jm}.$

In practice it is often of interest to identify differentially expressed genes. For example, it is important to assess whether the overall mean pattern of gene expression levels remains constant across all or pre-specified tissue groups. To do this, *HDTD* implements the testing methods proposed by Touloumis *et al.* (2015).

To estimate $\Sigma_{\text{R}}$ and $\Sigma_{\text{C}}$, shrinkage approaches are employed. These have been found to be extremely useful in constructing reliable gene networks (see Schäfer and Strimmer, 2005). The novel shrinkage covariance estimators derived in the Supplementary Material are statistically efficient and practical because they are invertible and easy to calculate regardless the number of genes and tissues. In addition, *HDTD* allows users to study correlation patterns of the genes or tissues by testing against known covariance structures (Touloumis *et al.*, 2014). The non-parametric nature of our analysis provides some robustness against non-normality.

## 3 Multiple tissue example

Melé *et al.* (2015) investigated variability in the human transcriptome across multiple tissues by analyzing RNA sequencing (RNAseq) data from the Genotype-Tissue Expression project. This project identified, among other things, genes whose expression signature characterized particular tissues. To accomplish this, Melé *et al.* (2015) used essentially all available tissue-samples from each of the 175 individuals by aggregating gene expression levels across the tissue tested and the remaining tissues (see §3.5 in Supplementary Material in Melé *et al.*, 2015). This approach does not acknowledge the tissue-wise correlation and consequently, this can affect the discovery of tissue-specific gene lists (Touloumis *et al.*, 2015). Since *HDTD* requires measurements from the same set of tissues across subjects, we considered a subset of this dataset including only the subjects (*N* = 11) with available RNAseq samples across all the most frequently collected tissues (skin, nerve, adipose, artery, lung, skeletal muscle, heart, blood and thyroid). A 44781 × 9 data matrix was created for each subject, with rows corresponding to genes, columns corresponding to the samples from the nine tissues and entries corresponding to the RPKM values. We use RPKM values for consistency with the original publication but we excluded genes where the sum of the RPKM values across the tissues was less than 0.1. To illustrate benefits when utilizing *HDTD*, we focused on two important inferential aspects: (i) study of the dependence structure among the nine tissues and (ii) corroboration of the gene signatures when the dependence between tissues is accounted for.

To study the tissue-specific variability, we estimated the corresponding covariance matrix Σ_c_ (Table S1 in the Supplementary Material). Blood was by far the most variable tissue (*SE* = 870.4), with SE at least four times that of the other tissues. To study the tissue-wise correlation, we calculated the correlation matrix from Σ_c_ (Table S2 in the Supplementary Material). We observed that lung, skeletal muscle, heart and thyroid were mildly correlated with each other (correlations ≥ 0.1), while the remaining tissues showed weaker strength of correlation. To investigate the statistical significance of our observation, we employed the sphericity test (Touloumis *et al.*, 2014) to all possible tissue pairs so as to identify correlated pairs of tissues. After applying an FDR correction, we failed to reject the sphericity hypothesis for the tissue pairs listed in Table S3 in the Supplementary Material. To summarize these results, there seems to exist a weak but statistically significant tissue-wise correlation pattern that needs to be considered when analyzing the gene expression pattern across tissues.

Melé *et al.* (2015) generated lists of genes that showed tissue-specific expression (Table S5 in Melé *et al.*, 2015). For a given tissue, we tested the hypotheses of conservation of the overall mean gene-expression levels of the corresponding genes-list between this tissue and any of the other eight, leading to a total of eight *P*-values, to which we applied an FDR correction. Failure to reject all hypotheses means that we do not have enough evidence that these genes are tissue-specific in their expression. After performing this analysis, we confirmed the validity of the tissue-specific gene-lists for skin, nerve, lung, skeletal muscle, heart and blood tissue. However, we failed to confirm that the overall mean gene-expression levels of the thyroid-specific gene-list is different in skeletal muscle (*P*-value = 0.782); that of the adipose-specific gene-list different in the skin (*P*-value = 0.105), and that of the artery-specific gene-list different in skin (*P*-value = 0.668), in adipose (*P*-value = 0.716), and in blood (*P*-value = 0.145). We also failed to reject the hypothesis that the mean gene-expression pattern for the artery-specific genes is simultaneously preserved across artery, skin, adipose and blood tissues (*P*-value = 0.412), which is in accordance with the pairwise tissue analysis. The difference in our conclusions compared to those in Melé *et al.* (2015) presumably arises because the methods in *HDTD* account for the presence of the tissue-wise correlation, regardless of its strength, a key inferential property that is not discussed by Melé *et al.* (2015).

## 4 Summary

Although *HDTD* was motivated by and illustrated using multi-tissue gene expression data, we emphasize that *HDTD* is suitable for analyzing other types of high-dimensional transposable data including single-cell transcriptomics data (see Lee *et al.*, 2014; Lovatt *et al.*, 2014) sampled from different tissues. In these studies, *HDTD* should lead to more robust inference since it accounts for both the gene- and tissue-wise correlation and is reliable for large numbers of cells without a dramatic increase in the computational cost.

## Supplementary Material

Supplementary Data
