# Supplementary material for: HDTD: analyzing multi-tissue gene expression data
Source: Bioinformatics. 2016 Jun 7;32(14):2193–5. doi: 10.1093/bioinformatics/btw224 (PMC4937203; doi:10.1093/bioinformatics/btw224)
Supplement: Supplementary Data [file supp_32_14_2193__index.html]

HDTD: Analyzing multi-tissue gene expression data — HDTD: analyzing multi-tissue gene expression data — HDTD: analyzing multi-tissue gene expression data — Supplementary Data 

# HDTD: analyzing multi-tissue gene expression data

## Supplementary Data

files

- Supplementary Data - pdf file
