# Supplementary material for: HDTD: analyzing multi-tissue gene expression data
Source: Bioinformatics. 2016 Jun 7;32(14):2193–5. doi: 10.1093/bioinformatics/btw224 (PMC4937203; doi:10.1093/bioinformatics/btw224)
Supplement: Supplementary Data [file supp_btw224_SupplementaryMaterial.pdf]

BIOINFORMATICS

Vol. 00 no. 00 2016
Pages 1–3

Supplementary Material for “HDTD: Analyzing multi-tissue gene expression data”

Anestis Touloumis [1,2*], John C. Marioni [1,3] and Simon Tavaré [1]

[1] CRUK Cambridge Institute, University of Cambridge, Li Ka Shing Centre, Robinson Way, Cambridge CB2 0RE, United Kingdom.
[2] Computing, Engineering and Mathematics, University of Brighton, Brighton, BN2 4GJ, United Kingdom.
[3] EMBL-European Bioinformatics Institute, Wellcome Genome Campus, Hinxton, Cambridge CB10 1SD, United Kingdom.

Received on XXXXX; revised on XXXXX; accepted on XXXXX

Associate Editor: XXXXXXX

STATISTICAL METHODS

A full description of the hypothesis testing procedures for the mean matrix and the covariance matrices can be found in Touloumis *et al.* (2015) and Touloumis *et al.* (2014), respectively. Herein, we derive the exact formulae for the shrinkage estimators of the two covariance matrices $\boldsymbol{\Sigma}_{\mathrm{C}}$ and $\boldsymbol{\Sigma}_{\mathrm{R}}$. To accomplish this, we extend the Stein-type covariance matrix estimation approach employed in Touloumis (2015) for estimating a single covariance matrix with vector-valued random variables to simultaneous estimation of two covariance matrices with matrix-valued random variables. For identifiability reasons, we impose the restriction $\mathrm{tr}(\boldsymbol{\Sigma}_{\mathrm{R}}) = r$ (see Touloumis *et al.*, 2014), where $\mathrm{tr}(\boldsymbol{A})$ denotes the trace of the square matrix $\boldsymbol{A}$, that is the sum of the diagonal elements of $\boldsymbol{A}$. It can be readily shown that the gene-wise variance scaling restriction $\mathrm{tr}(\boldsymbol{\Sigma}_{\mathrm{R}}) = r$ does not affect the gene- and column-wise correlation pattern.

To estimate the column covariance matrix $\boldsymbol{\Sigma}_{\mathrm{C}}$, first let

$$\boldsymbol{\Sigma}^{*}_{\mathrm{C}} = (1-\lambda_{\mathrm{C}})\frac{1}{Nr}\sum_{i=1}^{N}\boldsymbol{Y}_i^T\boldsymbol{Y}_i + \lambda_{\mathrm{C}}\mu_{\mathrm{C}}\boldsymbol{I}_c \qquad (1)$$

where $\boldsymbol{Y}_i = \boldsymbol{X}_i - \widehat{\boldsymbol{M}}$, $\widehat{\boldsymbol{M}}$ is the sample mean matrix, and where $\boldsymbol{I}_c$ is the identity matrix of size c. The optimal shrinkage intensity λ_{C} and the parameter μ_{C} are defined so as to minimise the expectation of $\mathrm{tr}\left[(\boldsymbol{\Sigma}^{*}_{\mathrm{C}} - \boldsymbol{\Sigma}_{\mathrm{C}})^2\right]$. This strategy leads to the closed form solutions

$$\lambda_{\mathrm{C}} = \frac{\frac{\mathrm{tr}(\boldsymbol{\Sigma}^2_{\mathrm{R}})}{r^2(N-1)}\left[\mathrm{tr}^2(\boldsymbol{\Sigma}_{\mathrm{C}}) + \mathrm{tr}(\boldsymbol{\Sigma}^2_{\mathrm{C}})\right]}{\frac{\mathrm{tr}(\boldsymbol{\Sigma}^2_{\mathrm{R}})}{r^2(N-1)}\left[\mathrm{tr}^2(\boldsymbol{\Sigma}_{\mathrm{C}}) + \mathrm{tr}(\boldsymbol{\Sigma}^2_{\mathrm{C}})\right] + \mathrm{tr}(\boldsymbol{\Sigma}^2_{\mathrm{C}}) - \mathrm{tr}^2(\boldsymbol{\Sigma}_{\mathrm{C}})/c}$$

and $\mu_{\mathrm{C}} = \mathrm{tr}(\boldsymbol{\Sigma}_{\mathrm{C}})/c$. Both λ_{C} and μ_{C} depend on the unknown parameters $\mathrm{tr}(\boldsymbol{\Sigma}_{\mathrm{C}})$, $\mathrm{tr}(\boldsymbol{\Sigma}^2_{\mathrm{C}})$ and $\mathrm{tr}(\boldsymbol{\Sigma}^2_{\mathrm{R}})$ which can be estimated by

$$T_{1N} = \frac{1}{rN}\sum_{i=1}^{N}\mathrm{tr}(\boldsymbol{X}_i^T\boldsymbol{X}_i) - \frac{1}{cN(N-1)}\sum_{i\neq j}\mathrm{tr}(\boldsymbol{X}_i^T\boldsymbol{X}_j),$$

$$\begin{aligned} T_{2N} = {} & \frac{1}{r^2N(N-1)}\sum_{i\neq j}\mathrm{tr}(\boldsymbol{X}_i^T\boldsymbol{X}_i\boldsymbol{X}_j^T\boldsymbol{X}_j) \\ & - \frac{2}{r^2N(N-1)(N-2)}\sum_{i\neq j\neq k}\mathrm{tr}(\boldsymbol{X}_i^T\boldsymbol{X}_i\boldsymbol{X}_j^T\boldsymbol{X}_k) \\ & + \frac{1}{r^2N(N-1)(N-2)(N-3)}\sum_{i\neq j\neq k\neq l}\mathrm{tr}(\boldsymbol{X}_i^T\boldsymbol{X}_j\boldsymbol{X}_k^T\boldsymbol{X}_l), \end{aligned}$$

and

$$\begin{aligned} T_{3N} = {} & \frac{T_{2N}^{-1}}{N(N-1)}\sum_{i\neq j}(\boldsymbol{R}_i^T\boldsymbol{R}_j)^2 \\ & - \frac{2T_{2N}^{-1}}{N(N-1)(N-2)}\sum_{i\neq j\neq k}\boldsymbol{R}_i^T\boldsymbol{R}_j\boldsymbol{R}_i^T\boldsymbol{R}_k \\ & + \frac{T_{2N}^{-1}}{N(N-1)(N-2)}\sum_{i\neq j\neq k\neq l}\boldsymbol{R}_i^T\boldsymbol{R}_j\boldsymbol{R}_k^T\boldsymbol{R}_l, \end{aligned}$$

respectively. Here, $\boldsymbol{R}_i$ denotes the vectorized form of $\boldsymbol{X}_i$ obtained by stacking the columns of $\boldsymbol{X}_i$ on top of one another. Following results in Touloumis *et al.* (2014), it can be shown that T_{1N}, T_{2N} and T_{3N} are consistent estimators of $\mathrm{tr}(\boldsymbol{\Sigma}_{\mathrm{C}})$, $\mathrm{tr}(\boldsymbol{\Sigma}^2_{\mathrm{C}})$ and $\mathrm{tr}(\boldsymbol{\Sigma}^2_{\mathrm{R}})$, respectively. Thus, we estimate λ_{C} with the consistent estimator

$$\widehat{\lambda}_{\mathrm{C}} = \frac{\frac{T_{3N}}{r^2(N-1)}\left(T_{1N}^2 + T_{2N}\right)}{\frac{T_{3N}}{r^2(N-1)}\left(T_{1N}^2 + T_{2N}\right) + T_{2N} - T_{1N}^2/c}$$

and we estimate μ_{C} with the consistent estimator

$$\widehat{\mu}_{\mathrm{C}} = T_{1N}/c.$$

*to whom correspondence should be addressed

© Oxford University Press 2016.

Finally, the shrinkage estimator of the column covariance matrix $\boldsymbol{\Sigma}_{\mathrm{C}}$ is the plug-in estimator of (1), that is

$$\widehat{\boldsymbol{\Sigma}}_{\mathrm{C}} = (1-\widehat{\lambda}_{\mathrm{C}})\frac{1}{Nr}\sum_{i=1}^{N}\boldsymbol{Y}_i^T\boldsymbol{Y}_i + \widehat{\lambda}_{\mathrm{C}}\widehat{\mu}_{\mathrm{C}}\boldsymbol{I}_c.$$

Next, to estimate the row covariance matrix $\boldsymbol{\Sigma}_{\mathrm{R}}$, let

$$\boldsymbol{\Sigma}^*_{\mathrm{R}} = (1-\lambda_{\mathrm{R}})\frac{1}{N\mathrm{tr}(\widehat{\boldsymbol{\Sigma}}_{\mathrm{C}})}\sum_{i=1}^{N}\boldsymbol{Y}_i\boldsymbol{Y}_i^T + \lambda_{\mathrm{R}}\boldsymbol{I}_r$$

where

$$\lambda_{\mathrm{R}} = \frac{\frac{1}{N-1}\frac{\mathrm{tr}(\boldsymbol{\Sigma}^2_{\mathrm{C}})}{\mathrm{tr}^2(\boldsymbol{\Sigma}_{\mathrm{C}})}\left[r^2+\mathrm{tr}(\boldsymbol{\Sigma}^2_{\mathrm{R}})\right]}{\frac{1}{N-1}\frac{\mathrm{tr}(\boldsymbol{\Sigma}^2_{\mathrm{C}})}{\mathrm{tr}^2(\boldsymbol{\Sigma}_{\mathrm{C}})}\left[r^2+\mathrm{tr}(\boldsymbol{\Sigma}^2_{\mathrm{R}})\right]+\mathrm{tr}(\boldsymbol{\Sigma}^2_{\mathrm{R}})-r}$$

minimizes the expected value of $\mathrm{tr}\left[(\boldsymbol{\Sigma}^*_{\mathrm{R}}-\boldsymbol{\Sigma}_{\mathrm{R}})^2\right]$. In a similar manner as with $\boldsymbol{\Sigma}^*_{\mathrm{C}}$, we can estimate λ_{R} with the consistent estimator

$$\widehat{\lambda}_{\mathrm{R}} = \frac{\frac{1}{N-1}\frac{T_{2N}}{T^2_{1N}}(r^2+T_{3N})}{\frac{1}{N-1}\frac{T_{2N}}{T^2_{1N}}(r^2+T_{3N})+T_{3N}-r}$$

and then use

$$\widehat{\boldsymbol{\Sigma}}_{\mathrm{R}} = (1-\widehat{\lambda}_{\mathrm{R}})\frac{1}{N\mathrm{tr}(\widehat{\boldsymbol{\Sigma}}_{\mathrm{C}})}\sum_{i=1}^{N}\boldsymbol{Y}_i\boldsymbol{Y}_i^T + \widehat{\lambda}_{\mathrm{R}}\boldsymbol{I}_r$$

as a shrinkage estimator of the gene covariance matrix $\boldsymbol{\Sigma}_{\mathrm{R}}$.

Derivation of the expectations of $\mathrm{tr}\left[(\boldsymbol{\Sigma}^*_{\mathrm{C}}-\boldsymbol{\Sigma}_{\mathrm{C}})^2\right]$ and $\mathrm{tr}\left[(\boldsymbol{\Sigma}^*_{\mathrm{R}}-\boldsymbol{\Sigma}_{\mathrm{R}})^2\right]$ relies on a matrix-variate normal model (Allen and Tibshirani, 2010) assumption for the matrix-valued random variables $\boldsymbol{X}_1,\ldots,\boldsymbol{X}_N$. However, under mild moment assumptions outlined in Touloumis *et al.* (2014) and by carrying out similar arguments as in Touloumis (2015), it can be argued that $\widehat{\boldsymbol{\Sigma}}_{\mathrm{R}}$ and $\widehat{\boldsymbol{\Sigma}}_{\mathrm{C}}$ are robust to departures from the normality assumption. Therefore, *HDTD* implements nonparametric estimation and testing procedures that can be used beyond the scope of the matrix-variate normal model.

MULTIPLE TISSUE EXAMPLE

The "GTEx Analysis V4" gene-expression RPKM values were available for downloading from http://www.gtexportal.org/home (last assessed on March 19, 2016). The subsamples were then identified using the accompanying annotation files.

The nine tissue samples in each subject-specific data matrix were ordered as skin (sun exposed), nerve, adipose (subcutaneous), artery (tibial), lung, skeletal muscle, heart (left ventricle), blood and thyroid. The estimated tissue covariance matrix $\widehat{\boldsymbol{\Sigma}}_{\mathrm{C}}$ is given in Table 1 and the resulting tissue-wise correlation matrix calculated from $\widehat{\boldsymbol{\Sigma}}_{\mathrm{C}}$ is given in Table 2 (both Tables are presented using a two-letter abbrevation for the tissues). From these two matrices, we can infer the tissue-specific variability by inspecting the diagonal elements of $\widehat{\boldsymbol{\Sigma}}_{\mathrm{C}}$ and the correlation of all possible tissue pairs. For example, the estimated variances for the lung and the skeletal muscle are 18244.2 and 27828.3 respectively, while their correlation is 0.13, that is the element (5,6) in Table 2. By comparing the diagonal elements of $\widehat{\boldsymbol{\Sigma}}_{\mathrm{C}}$, we can deduce that blood was the most variable tissue ($SE=\sqrt{757511.9}=870.4$).

Table 3 displays the adjusted p-values that are greater than 0.05 when testing the sphericity hypothesis for all possible pairs of tissues. Although we rejected the sphericity hypothesis for the majority of tissue pairs, it is worth noting that the skin, the adipose and the lung tissue appear to form a network of mutually uncorrelated tissues with equal variance (p-value= 0.082).

Table 4 displays the adjusted p-values when testing the statistical significance of the tissue-specific gene-lists provided by Melé *et al.* (2015). For a given tissue, small adjusted p-values across the remaining eight tissues suggest that the differential expression of the gene-list under study is tissue-specific. From our analysis, the tissue-specificity of the artery-, adipose- and thyroid-specific gene lists provided by Melé *et al.* (2015) was not supported.

The analysis in the main text and the Supplementary Materials can be reproduced by executing the commands in the R script.

REFERENCES

Allen, G.I. and Tibshirani, R. (2010). Transposable regularized covariance models with an application to missing data imputation. *The Annals of Applied Statistics*, **4**, 764–790.

Melé, M., *et al.* (2015). The human transcriptome across tissues and individuals. *Science*, **348**, 660–665.

Touloumis, A., Marioni, J.C. and Tavaré, S. (2014). Hypothesis testing for the covariance matrix In high-dimensional transposable data with Kronecker product dependence structure. *Submitted. arXiv:1404.7684v2*.

Touloumis, A., Tavaré, S. and Marioni, J.C. (2015). Testing the mean matrix in high-dimensional transposable data. *Biometrics*, **71**, 157–166.

Touloumis, A. (2015). Nonparametric Stein-type shrinkage covariance matrix estimators in high-dimensional settings. *Computational Statistics and Data Analysis*, **83**, 251–261.

Table 1. The estimated tissue-wise covariance matrix $\widehat{\Sigma}_{\mathrm{C}}$.

	SK	NE	AD	AR	LU	SM	HE	BL	TH
SK	19122.9	576.1	263.6	519.7	543.9	1176.7	2995.3	208.3	1159.8
NE	576.1	16882.4	554.0	860.1	751.6	1847.0	2723.1	138.3	1050.0
AD	263.6	554.0	17523.0	461.8	416.6	741.5	1286.6	159.2	617.4
AR	519.7	860.1	461.8	17070.6	796.2	1904.7	2442.6	300.5	1190.7
LU	543.9	751.6	416.6	796.2	18244.2	2824.8	5641.3	3497.2	1963.9
SM	1176.7	1847.0	741.5	1904.7	2824.8	27828.3	13027.8	348.6	3589.7
HE	2995.3	2723.1	1286.6	2442.6	5641.3	13027.8	53880.8	1231.6	9565.9
BL	208.3	138.3	159.2	300.5	3497.2	348.6	1231.6	757511.9	4730.4
TH	1159.8	1050.0	617.4	1190.7	1963.9	3589.7	9565.9	4730.4	20444.9

Table 2. The estimated tissue-wise correlation matrix based on $\widehat{\Sigma}_{\mathrm{C}}$.

	SK	NE	AD	AR	LU	SM	HE	BL	TH
SK	1.00	0.03	0.01	0.03	0.03	0.05	0.09	0.00	0.06
NE	0.03	1.00	0.03	0.05	0.04	0.09	0.09	0.00	0.06
AD	0.01	0.03	1.00	0.03	0.02	0.03	0.04	0.00	0.03
AR	0.03	0.05	0.03	1.00	0.05	0.09	0.08	0.00	0.06
LU	0.03	0.04	0.02	0.05	1.00	0.13	0.18	0.03	0.10
SM	0.05	0.09	0.03	0.09	0.13	1.00	0.34	0.00	0.15
HE	0.09	0.09	0.04	0.08	0.18	0.34	1.00	0.01	0.29
BL	0.00	0.00	0.00	0.00	0.03	0.00	0.01	1.00	0.04
TH	0.06	0.06	0.03	0.06	0.10	0.15	0.29	0.04	1.00

Table 3. List of adjusted p-values > 0.05 from applying the sphericity test to all possible tissue pairs.

Tissue 1	Tissue 2	Adjusted p-value
skin	adipose	0.2837
skin	lung	0.5914
skin	thyroid	0.1099
nerve	adipose	0.0588
adipose	artery	0.7992
adipose	lung	0.2724

Table 4. The adjusted p-values from testing the significance of tissue-specific gene lists (rows) to each one of the remaining tissues (columns).

Tissue-Specific Gene List	SK	NE	AD	AR	LU	SM	HE	BL	TH
SK		0.000	0.000	0.000	0.000	0.000	0.000	0.000	0.000
NE	0.000		0.000	0.000	0.000	0.000	0.000	0.000	0.000
AD	0.105	0.043		0.001	0.000	0.000	0.000	0.000	0.003
AR	0.668	0.716	0.007		0.000	0.000	0.000	0.148	0.000
LU	0.037	0.000	0.000	0.001		0.000	0.000	0.007	0.000
SM	0.000	0.000	0.000	0.000	0.000		0.000	0.000	0.000
HE	0.000	0.000	0.000	0.000	0.000	0.000		0.000	0.000
BL	0.000	0.000	0.000	0.000	0.000	0.000	0.000		0.000
TH	0.000	0.000	0.000	0.000	0.000	0.781	0.000	0.000	
